# Treating Different Diseases With the Same Method—A Traditional Chinese Medicine Concept Analyzed for Its Biological Basis

**DOI:** 10.3389/fphar.2020.00946

**Published:** 2020-06-26

**Authors:** Xing Zhai, Xi Wang, Li Wang, Linlin Xiu, Weilu Wang, Xiaohan Pang

**Affiliations:** ^1^ School of Management, Beijing University of Chinese Medicine, Beijing, China; ^2^ College of Humanities，Beijing University of Chinese Medicine, Beijing, China; ^3^ College of Traditional Chinese Medicine, Beijing University of Chinese Medicine, Beijing, China

**Keywords:** treating different diseases with the same method, traditional Chinese medicine, information extraction, complex network, TCMIP v2.0

## Abstract

**Introduction:**

The fundamental theory of traditional Chinese medicine (TCM) implies that when different diseases have the same pathogen, the syndromes of these individual diseases will be the same. “Treating different diseases with the same method” is a TCM principle suggesting that when different diseases have similar pathological changes during different stages of their development, the same method of treatment can be applied. Our study aims to analyze the concept “treating different diseases with the same method” from a molecular perspective, in order to clarify its biological basis and to objectively standardize future TCM syndrome research.

**Objective:**

The TCM syndromes Qi deficiency and blood stasis have similar pathogenesis in relation to coronary heart disease (CHD) and stroke. We aim to use big data technology and complex network theory to mine the genes specifically relevant to these TCM syndromes. This study aims to explore the correlation between the biological indicators of CHD and stroke from a scientific perspective.

**Methods:**

Mining the relevant neuroendocrine-immune (NEI) genes by means of gene entity recognition, complex network construction, network integration, and decomposition to categorize relevant syndrome terms and establish a digital dictionary of gene specifically related to individual diseases. We analyzed the biological basis of “treating different diseases with the same method” from a molecular level using the TCMIP v2.0 platform in order to categorize the TCM syndromes most relevant to CHD and stroke.

**Results:**

We found 46 genes were involved in the TCM syndromes of Qi deficiency and blood stasis of CHD and stroke. The same genes and their molecular mechanism also appeared to be in close relation to inflammatory response, apoptosis, and proliferation.

**Conclusion:**

By using information extraction and complex network technology, we discovered the biological indicators of the TCM syndromes Qi deficiency and blood stasis of CHD and stroke. In the era of big data, our results can provide a new method for the researchers of TCM syndrome differentiation, as well as an effective and specific methodology for standardization of TCM.

## Preface

“Treating different diseases with the same treatment” is an important part of the fundamental theory of TCM, embodying the spirit of “treatment by syndrome differentiation”. A similar point of view can be seen in modern medicine where it is academically accepted that certain disease mechanisms lead to various diseases. For example, insulin resistance exists in obesity, diabetes, dyslipidemia, and other metabolic diseases. Another example is that the inflammatory response mechanism can be found in infections, atherosclerosis, glomerulosclerosis, hypertension, and ischemia-reperfusion of heart and brain. The knowledge that certain diseases have shared pathological mechanisms has a significant impact on the study of pathology both in modern medicine and TCM. From their respective perspectives both TCM and modern medicine acknowledge the importance of shared pathological mechanism of diseases. Therefore, the concept of “treating different diseases with the same method” can be seen as a focal point of the integration and unification of TCM and modern medicine (Xuezhong et al., 2004).

In the era of big data, the acknowledgment of TCM theory is closely related to informatics. The standardization and digitalization of TCM diagnosis need information and big data to improve the research. Information technology is needed to make a breakthrough in the fundamental research of TCM syndrome differentiation and biology. Getting research results published is common procedure for any scholar to have their research known and acknowledged, TCM is no exception. To this day, a large number of research documents related to TCM are stored in WOS, PubMed, and other international medicine databases. However, only a small amount of these papers and their knowledge can be found by the means of Artificial Intelligence (AI) and algorithms and has to be individually searched and found by humans. It will be of great significance for the medical society to discover the relationship between TCM syndrome differentiation and modern molecular biology. We aim to discover and compare a vast number of medical journals and texts to explore “treating different diseases with the same method” from the perspective of microbiology by using information technology (including machine learning, text mining, complex network, etc.)

To begin our research of “treating different diseases with the same method”, we chose diseases and syndromes with a high incidence rate, complex treatment and which have a major impact on the health of the general population. With the rapid development of the Chinese national economy peoples living conditions and lifestyles have changed dramatically, ultimately resulting in a shift of the most common diseases. The incidence rate of coronary heart disease (CHD) and stroke is increasing. According to the China Health and Family Planning Statistical Yearbook, released by the national health and family planning commission, stroke and CHD have become the leading cause of death among Chinese citizens. Additionally, a WHO survey points out that the incidence rate of stroke is ranked first among diseases in China and is now trending toward younger parts of the population. How to effectively prevent and treat these two diseases has become a key point in the medical field.

Qi deficiency and blood stasis are important syndromes in the diagnosis and treatment of TCM. These two TCM syndromes are common in CHD, stroke, tumor, hypertension, cerebral infarction, and other multi-system diseases. To treat the above diseases, TCM doctors use prescriptions to invigorate Qi and activate blood. For example, *Buyang Huanwu Decoction* is a commonly used formula for the treatment of the syndromes of Qi deficiency and blood stasis, which in turn has a good effect on CHD and stroke. This principle of treating different diseases in their respective stage of development with the same method principle is the essence of “treating different diseases with the same method”. In this paper, we chose the common mechanism of CHD and stroke as our focal point to discover the biological basis of “treating different diseases with the same method”. This paper is divided into four chapters. Chapter 1, Introduction of research in China and abroad. Chapter 2, Introduction of data and methods used in this study. Chapter 3, Study results. Chapter 4, Summarization of research and suggestions for future research.

## Literature Review

### Information Extraction (IE) and Its Applications in the Biomedical Field

IE refers to the process of extracting, integrating and processing relevant information from existing data in order to store the specific information in a new data structure for future use and consultation (Bin, 2002). In IE, the uttermost important is to discover and extract the relationship between various entries. At present, relationship extraction generally applies to relationship extraction in dictionaries, as part of pattern matching and by means of machine learning. The dictionary based approach matches the words in the corpus with those in the professional dictionary in order to identify the words and/or their relationships. By matching the relevant literature and gene dictionaries related to Qi deficiency and blood stasis syndromes of CHD, Zhao (Xing et al., 2015) identified the genes most relevant to these syndromes. Using relationship extractions based on dictionaries is simple and fast, but in the era of big data, the speed that information updated is even faster leading to dictionaries lacking behind the current information. Thus it can be understood that this method is not suitable for scenarios where the data of dictionaries is updated on a regular basis.

Relation extraction of pattern matching is based on the observation and analysis of entries by linguistic experts to define the specific rules and extract the correlation of biomedical entries. For example, in 2001, Ono et al. (2001) created an extraction model base on relations between protein-protein interaction (PPI) relationships by observing regular expressions, shallow syntactic patterns, and pattern matching method. Although methods based on pattern matching can extract the relationship between biomedical entities more accurately, it is difficult to identify the relationships outside of the defined set of rules. Therefore, the accuracy is often high but at the same time the recall rate is low.

Based on machine learning, the relevant features and parameters are calculated from the sample data and following used to identify and establish the new model. Machine learning can be further divided into eigenvector based methods and kernel based methods. Feature vector based method, which belongs to supervised machine learning and eigenvector based method, is a commonly used machine learning method applied in biomedical entity relationship extraction. Feature vector based method is used to change the relation of linguistic information into plane features, and following to create a high-dimensional map in vector space. This is done in order to translate information of the natural language into recognizable vector classifiers. For example, using support vector machine (SVM) models Xiao et al. (2005) extracted PPI relationship by applying a combination of lexical, syntactic, and semantic features in combination with maximum entropy classifiers. Although feature vector based relation extraction method is flexible and has good performance, the spectrum of feature selection often directly determines the extent of relation extraction. At the same time, the quantity and quality of labeled corpus usually determines the performance of extraction. The core function is used to calculate the similarity of two candidate relationship instances in potential vector space. For example, Bunescu and Mooney (2005a) proposed the idea of applying kernel function method to extract protein relations. Based on kernel function they managed to isolate PPI relationships by extracting the dependency pathways between specific proteins. The kernel based method has better performance because it has more flexibility to extract correlating information between entities. However, per definition kernel function has a direct impact on the result of relation extraction. It can be seen that there is a common problem in relation extraction methods based on machine learning. It gives rise to similar issues, whether using eigenvector or kernel function for relation extraction. Due to the need of large quantity of labeled corpora the quality of these functions will deteriorate along with the quality of the labels.

### Use of Complex Network in Biomedical Research

The complex network theory is a method based on graph theory and complex system theory. Large-scale nodes, complex network structures, and dynamic spatiotemporal evolution of networks are the main characteristics of complex network. They coincide with the characteristics of data complexity, diversity, and the dynamic changes of informatics. Applying the theory and method of complex network to data analysis helps analysts to pinpoint what exact problems should be researched, and as a whole to improve the efficiency of their analytic tasks.

Complex network theory is not only applied to climate, politics, economy, society, military, and management, but also widely used in biomedical research. At present, the research of complex network in the biomedical field mainly focuses on the location of disease gene, disease-related sub-network recognition, network-based disease control research and drug target predictions. For example, Lim et al. (2006) used pathway information to discover pathogenic proteins. Deng et al. (2004) predicted a drug target method based on network topology. Artzy-Randrup et al. (2004) constructed the pin sub-network of Huntington's disease (HTT) and by these means discovered a new HTT pathogenic gene

Regarding TCM research, Li (Li et al., 2007) applied the information technology of text mining to prove that “disease” and “syndrome” have the possibility of a “disease syndrome combination” at the level of biological networks. Zhou et al. (Xuezhong et al., 2004) processed and integrated the database of TCM literature to find the gene related to kidney Yang deficiency and thereby provided an effective way to understand the function of this specific gene and its significance regarding the TCM syndrome. From the perspective of “interaction network function”, Li (Shao, 2007) explored and established the relevant method to apply computational system biology on TCM. He discovered through the excavation and research of the TCM syndromes of “cold and heat”, diseases, and prescriptions that the biological network of somatic angiogenesis is closely related to the TCM concept of” collateral disease”. Ding et al. (Fan et al., 2014) used network analysis method to study *Buyang Huanwu Decoction* from three aspects: chemical composition, target tissue, and related diseases to discover its potential in cancer treatment.

Since the 1970s, modern medicine has recognized the concept and interconnection of the neuro-endocrine-immune (NEI) systems. In 1973, Isaković and Janković (1973) firstly proposed the relationship between these three systems by injuring different parts off rat brains with bilateral symmetrical electrolysis and following observing their effects on lymphoid organs. The discovery and acknowledgment of NIE coincide with the overall concept advocated by TCM for thousands of years, and to a certain extent, it validates the theory of TCM, that has been developed through millennial of clinical practice. In 1990, Wang (Lan and Yusheng, 1990) firstly drew parallels between NEI network and TCM theory. Afterward, more scholars have researched the connection between TCM theory and NEI network, such as the study of “cold and heat” syndromes (Ma et al., 2010) and the study of therapeutic mechanism of acupuncture (Ding et al., 2013). The key of “treating different diseases with the same method” is that although diseases are different, the syndromes can be the same. By using NEI network to explore the correlation between biological indicators of different diseases, we got a deeper understanding of TCM which assists to its modernization and internationalization.

Through the use of information technology in biomedical science, this TCM research has achieved good results. We still see the need for improvements. The main problems are as follows: 1) although there are many experiments of TCM using information technology, modern medicine and molecular biology most of them lack the effective combination of the mathematical modeling of precise result mining. This in turn leads to the biological significance of TCM syndromes and diseases being undiscovered. In other words, there are no clear indicators of the correlation between the perspectives of macro biology and microbiology in the research. 2) In the current research on biological networks the constructed networks are large and complex in scale. Due to the complexity of the network indicators, the results are difficult to analyze. There is no known method to compress networks and define the similarity between nodes ones the networks are compressed. We proposed to apply machine learning, information extraction and complex network technology to construct gene networks based on TCM syndromes, as well as use data mining by combining decomposition and combination throughout these networks. Our hypothesis is that by using these technologies to discover common genetic nodes through calculations, analysis and mining it will be possible to discover the genes specifically related to the syndromes of Qi deficiency and blood stasis of CHD and stroke.

Mesh (Medical Subject Headings) technology was used to mine the specific genes of NEI throughout PubMed database to construct the biological network. We also explored the mechanism of the concept “treating different diseases with the same method” from the perspective of microbiology. This has deepened the understanding of the theoretical system of TCM syndrome differentiation, and promoted the studies of TCM syndromes.

## Materials and Methods

This article was based on biomedical literature downloaded from http://www.ncbi.nlm.nih.gov/entrez/query.fcgi, including the two target syndromes. This was chosen due to vast PubMed database managed by the United States National Library of Medicine and the National Institutes of Health, providing free catalogs of biomedical papers worldwide. With the increasing internationalization of TCM research, the biomedical literature in PubMed contains a large number of articles related to Traditional Chinese medicine (A total of 148,000 articles related to Traditional Chinese medicine dated from 2010 to 2020 found by searching the keywords “*Traditional Chinese medicine*”, “*herbal medicine*”, “*aspiration*” and “*prescriptions*”). These articles are valuable to TCM scientific research and innovation, TCM technological innovation and the understanding of the current situation of TCM clinical practice in and outside of China. The free and easy access to vast biomedical literature related to TCM and modern medicine is why we chose PubMed as our data source. In order to precisely mine the genetic data of the two target syndromes, and to improve the accuracy of the data mining, the author proposed a data mining model based on the combination of “network decomposition” and “symptom combination” to mine the NEI genetic data in PubMed database (Xing et al., 2015; Liu et al., 2019). Then, the author combined the results of the two methods to draw a final conclusion. The methods of mining are described below.

### Network Decomposition

**Figure 1:**
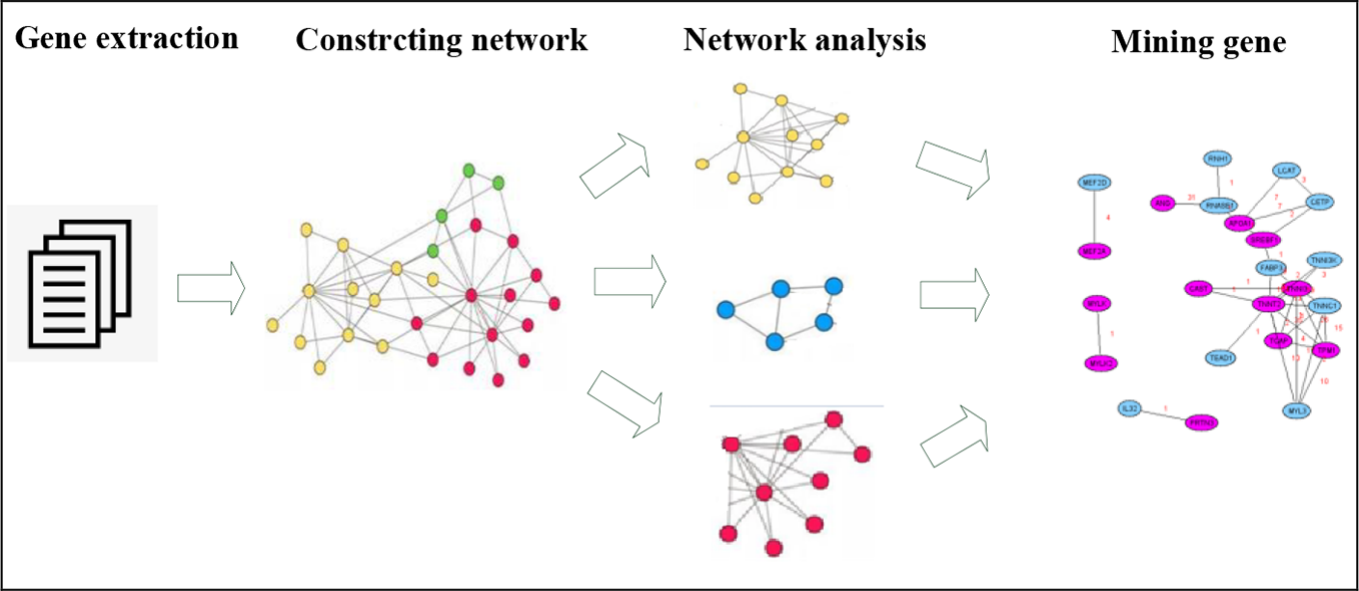
Flow chart of network decomposition method.

In order to reduce the complexity of network analysis, the network was divided into sub networks according to the network categories and the specific genes and their functions. Each sub network was individually analyzed and the results of each sub network were combined to find the genes related to the target syndromes. See **Figure 1**.

#### Data Collection

**Figure 2:**
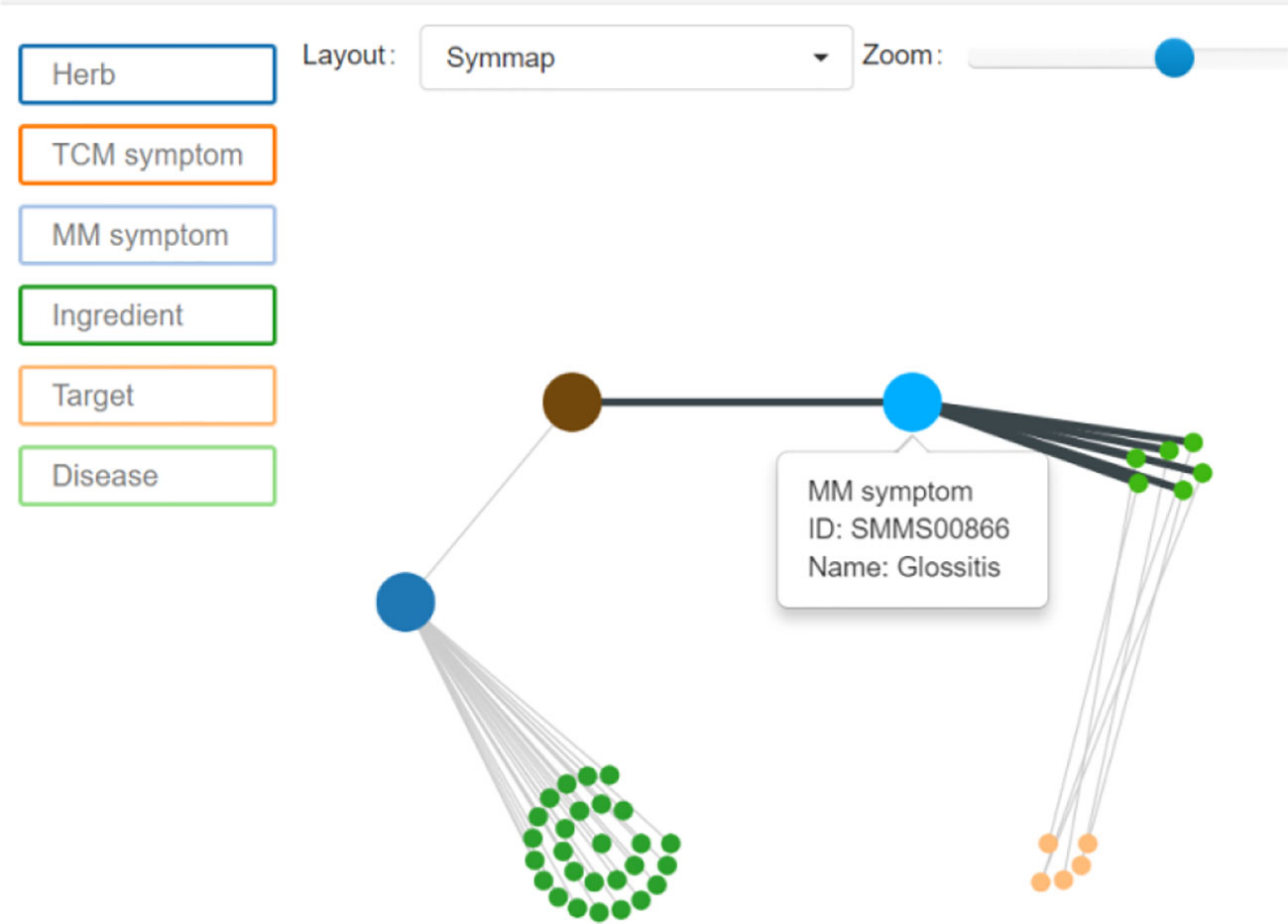
Symmap query interface.

According to the *Guidance Principle of Clinical Studies of New Drug of Traditional Chinese Medicine* (Xiao-yu, 2002), the terms of the target syndromes were selected. Then, two TCM experts weighted the rationality of these terms according to their personal clinical experiences to decisively determine the terms to be used in this paper. As the TCM terms chosen were all in Chinese and PubMed database in English, it was necessary to translate them into English. However, due to the specificity of TCM terminology many translated terms could not be searched in PubMed. Therefore, it was necessary to convert these TCM terms into corresponding modern medicine terminology. In this paper, Symmap database was used to finalize the translation of TCM terms into modern medicine terms. Symmap database (Wu et al., 2018) was developed by Chen Jian-xin, Professor of Beijing University of Chinese Medicine. Through Symmap and its classifications of internal molecular mechanism and external symptom, the database charted 1,717 TCM symptoms and allocated them to 961 modern medicine symptoms. For example, “red tongue” of TCM diagnosis is translated through Symmap database into “Glossitis” of western medicine (**Figure 2**).

The key terms were loaded into PubMed database according to the search format. We searched the Mesh subject terms and downloaded the literature summary. Following we downloaded the documents and converted them into a unified formation order to generate the corpus intended for mining.

#### Gene Information Extraction

**Figure 3:**
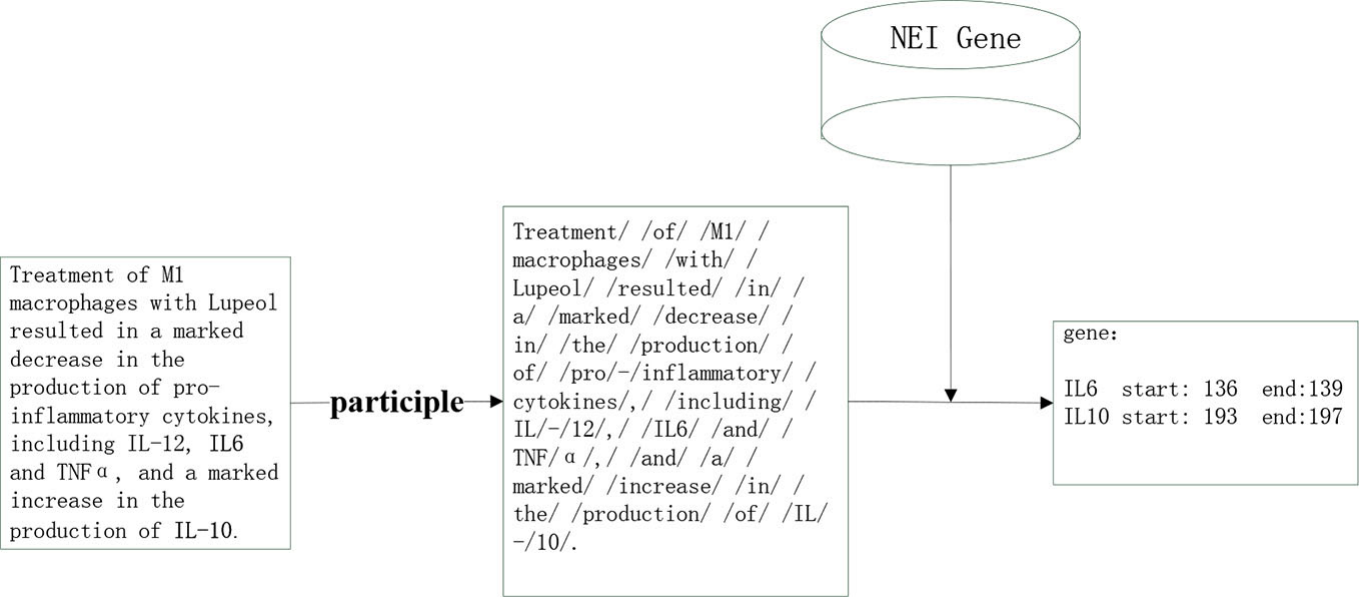
Gene entity recognition.

According to the basis of this paper, the entity of NEI genes that needed extraction from the literature was relatively defined and had little change. For improvements of efficiency and accuracy of entity extraction we used a dictionary matching method. The downloaded documents were pre-processed, and following divided into independent sentences that were further segmented by using the *Jieba* word, a python segmentation package. After this process the separated words were matched with the key words of the NEI gene Dictionary. The NEI gene dictionary was downloaded from dbNEI (http://bioinfo.au.tsinghua.edu.cn/dbNEIweb/) A total of 1,435 NEI genes were downloaded and used for comparison (**Figure 3**).

Gene relationship extraction is used to identify the possible gene relationships in literature abstracts (In this paper it mainly refers to the gene relationships recognized in the abstracts). We found the relationships between genes and understood their potential usage through analysis and identification of the specific genes and their interrelations. We used sparse multi-instance learning algorithm (SMIL) (Wu et al., 2018) to extract gene relationships based on the summaries of previous relationship extraction algorithms. This approach can be applied to relationship extraction. By assessing whether or not a specific group of genes have interactions, and whether or not these interactions are of identical genes.

In this study, each grouping contained multiple relationships. If the relationship between two or more genes were observed, then the group was labeled positive. If no relationships between genes were observed, the package was labeled negative. E.g. A study has two genes that may be mentioned several times throughout the abstract, but generally speaking a specific relationship will only be mentioned once. Therefore, the information regarding these two specific genes will be relative sparse. We decided to use SMIL to extract the genetic relationship and avoid overlooking important information in the literature abstracts acquired from PubMed.

In addition to the data packages, “knowledge base” and “file data” are needed to extract relationships using SMIL algorithm. “File data” is a data source that contains data relations. “Knowledge base” has the ability to replace manual labeling of existing relationships. Therefore, the “knowledge base” must contain the exact same relationships as the relationships examined for extraction.

There are many industry databases about the relationship between genes in the field of bioinformatics. Therefore, to obtain the industry knowledge base was easy. After the comparative analysis of these specific gene databases, we finally decided to build a knowledge base from the string database. String database (http://string-db.org/) can analyze protein-protein interaction and includes 5,090 organisms. It not only contains the protein-protein interaction data known through validated experiments, but the database also includes predictions of unknown protein-protein interaction. We copied and pasted all NEI genes into the string database through the “multiple proteins by names/identifiers” option, selected the set species as Homosapiens before beginning the search. The outcome of the protein relationship search was 823 nodes, 7,616 edges and the relationship values of each paired protein. We then downloaded the protein relationship data and used it to construct the knowledge base which contains the relationship between all NEI genes. The method of constructing the instance package is shown in algorithm 1. When two genes appear in the same paper abstract the whole text is traversed through the algorithm and the two genes added to a specific bag. If the specific gene relationship in bag exists in STRING knowledge base, the bag is flag as 1, otherwise as 0. We carried out this task until all the gene relationships in the knowledge base were mapped.

The algorithm used for word bag model generation is as follows (Lamurias et al., 2017):


function generate_bag (corpus,string)
    begin
    bags=[]
    for abstract in corpus do
    begin
     for gene in abstract do
    begin
       bag= (gene_i_,gene_j_)
       if bag not in bags then
       begin
          if bag in string then
              bag.flag=1
          else
              bag.flag=0
       end
        bags.add(bag)
       end
    end
    retrun bags
end


#### Network Analysis

The adjacency matrix was constructed based on the gene relationship mined by SMIL method and following converted into Pajek recognized.net file. The file was imported into Pajek software to generate a visualized gene network map. We applied the method of “decomposition integration” because the complexity of the genetic network. This method was used to divide the genetic network into sub networks (community nodes), that in turn were separately analyzed. Finally, the results of each sub network were recombined to analyze the results of the whole network. Referring to the results of Yi's research (YIDH, 2017), we calculated the community node centrality index, subnet weight index, standardized weight, and node genetic weight index. This was done according to the following formulas:

(formula 1) CMI =CD*CB*(1/CC)+CE

(formula 2) SW= N+ZJW

(formula 3) ZSW=(SW-MIN)/(MAX-MIN)

(formula 4) GW=CMI+ZSW

(CMI) comprehensive measurement indicator; (CD)centrality degree; (CB) betweenness centrality; (CC) closeness centrality; (CE) centrality eigenvector; (SW) subnet weight; (ZSW) standardized weight of SW; (N) number of nodes in subnet; (ZJW) The correlation between subnet function and the problem to be studied problem, which is scored by experts; (GW) gene weight.

### Combination Method

**Figure 4:**
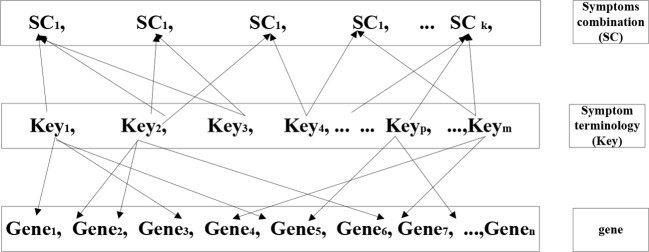
Schematic diagram of combination method.

Combination method is an analytic method used to combine equations and set aside variables to discover a unified value. We assumed that multiple genes would correspond to each individual symptom (key), and that multiple symptoms would in turn correspond to one TCM syndrome. We investigated the most relevant genes (**Figure 4**) through the research concept of “gene symptom syndrome”. The key words of target syndromes in modern medicine were regarded independently. Firstly, we discovered the genes related to the modern medicine syndrome, and following referred the data to the standard of TCM diagnosis for re-matching. As a final step we uncovered the specific gene which existed in each combination. This specific gene is the key gene and the result of these mentioned methods.

#### Search for Symptom Specific Gene

The search for symptom specific gene was done by the use of GenCliP software. GenCliP is a literature mining tool that can be used to form a list of genes with the use of keywords. These lists were then extracted by the software from their related literature and then manually verified. GenCliP displays specified genes and keywords mentioned in literature for manual association verification (Huang et al., 2008). We used the software to input all NEI genes in the “Upload Gene List”, and input each target syndrome and their symptom into the search box “Word Related Gene Search”. This was done to explore the NEI gene related to each specific symptom, and to define the symptom and the accompanying gene as “Abstract”. The results of these searches included two parts: “Gene” and “Hit”. “Gene” refers to the name of the searched gene, “Hit” refers to the number of documents in which the gene and the correlated searched symptom have appeared simultaneous. This concept can also be understood as the “weight of the gene”.

#### Symptomatic Combination

In TCM each disease and syndrome are composed of many symptoms, and these symptoms can be further divided into primary and secondary symptoms. Under the guidance of TCM experts, we combined the individual symptoms according to the TCM diagnostic standards to better reflect the essential characteristics of the disease related to this study. The TCM experts divided the target syndromes into three parts: main syndrome, Qi deficiency syndrome and blood stasis syndrome. According to the TCM diagnosis standard, experts combined the symptoms of these three sections in different models to extract the gene information of each individual combination. We used this information to plot the intersection of the symptom combinations, and to screen out the genes existing in each combination. Finally, combining the method of Li (Li et al., 2007) and the experience of TCM experts, we discovered the core genes of each individual target syndrome. We following compared the genes of two respective target syndromes by “combination” and “decomposition” methods to extract the relevant genes.

## Research Results

### Results of “Decomposition Method”

We retrieved the relationships between TCM symptoms and modern medicine symptoms based on the SymMap database. **Tables 1** and **2** show translation from Chinese of symptom keywords in English based on TCM terminology. These keywords were used for literature search in PubMed.

**Table 1:** Key words of Qi deficiency and blood stasis syndrome of coronary heart disease.

TCM symptoms	胸痛, 胸闷, 气短,心悸, 乏力, 畏寒/肢冷, 自汗, 不寐, 舌淡紫, 偏瘫, 瘀斑, 头晕目眩
Symptoms of modern medicine	Chest pain, chest heaviness, respiratory abnormality, palpitation, lassitude, typhoid fever/monoparesis/chills, sweating, insomnia, tongue disorder, hemiplegia, ecchymoses, dizziness

**Table 2:** Key words of Qi deficiency and blood stasis syndrome of stroke.

TCM symptoms	头晕目眩, 头痛, 易怒, 肢体麻木, 痰多, 气短, 乏力, 自汗, 便秘, 口干口渴, 舌质红, 舌质暗, 半身不遂, 口舌歪斜, 面色淡白, 瘀斑
Symptoms of modern medicine	Dizziness, headache, irritability, numbness of extremity, sputum, respiratory abnormality, lassitude, perspiration, constipation, xerostomia, glossitis, tongue disorder, hemiplegia, stroke, ecchymoses

**Figure 5:**
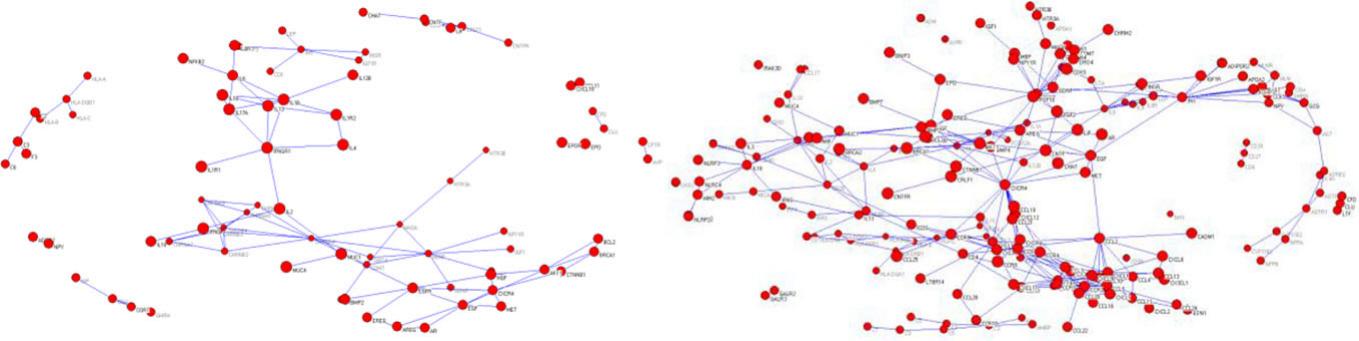
Gene network of (left) Qi deficiency and blood stasis in coronary heart disease and (right) Qi deficiency and blood stasis in stroke.

We used these keywords for Mesh retrieval in PubMed database. A total of 85,733 documents related to syndromes of Qi deficiency and blood stasis of CHD and 96,038 documents related to Qi deficiency and blood stasis syndromes of stroke were retrieved. We downloaded the documents (including author, title, key words, abstract, and so forth) and used them to generate the document library in XML format. A total of 84 NEI genes relevant to Qi deficiency and blood stasis syndrome of CHD and 199 NEI genes relevant to Qi deficiency and blood stasis syndrome of stroke were extracted by the mining method shown in *Gene Information Extraction*. Through the relationship recognition method shown in *Gene Information Extraction*, 109 gene pairs relevant to Qi deficiency and blood stasis syndrome of CHD and 313 gene pairs relevant to Qi deficiency and blood stasis syndrome of stroke were identified. The adjacency matrix of gene relationship was constructed and converted into a.net file recognized by Pajek software to generate a visualized gene network diagram (**Figure 5**).

**Figure 6:**
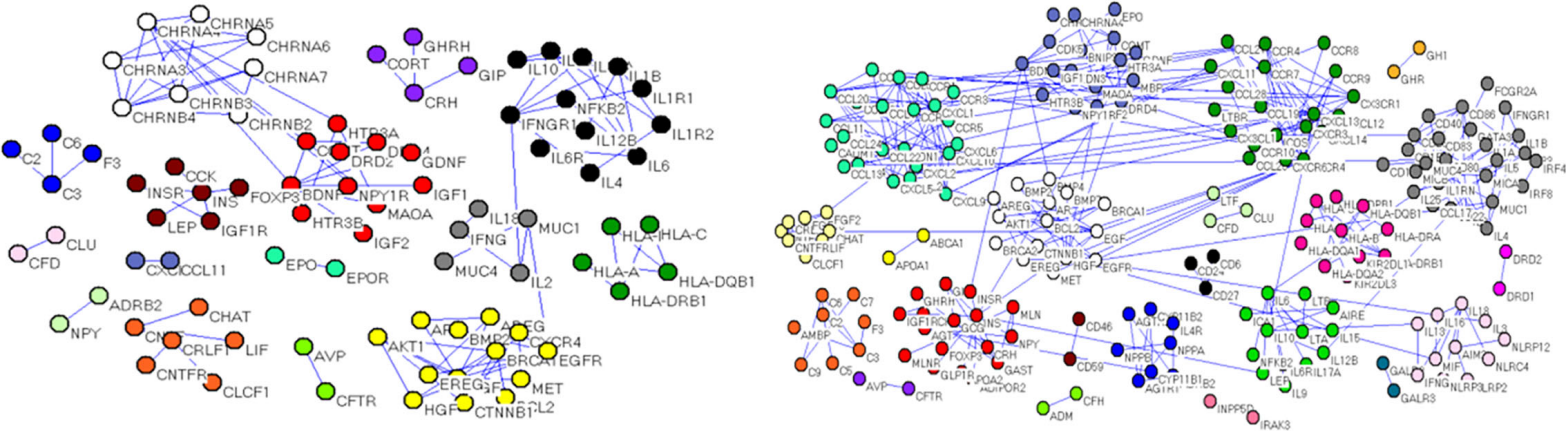
Gene sub network divisions, (left) Qi deficiency and blood stasis in CHD and (right) Qi deficiency and blood stasis in stroke.

Calculations of four centrality indexes per network were made from the gene nodes, and then, according to Formula 1 the comprehensive measurement value of the centrality index was calculated. As a final step each of the two networks were individually divided into sub networks according to the community division algorithm of complex network. The results showed that the genetic network of Qi deficiency and blood stasis syndromes of CHD was divided into 14 sub networks (left of **Figure 6**). Among them, there were three large communities with more than 10 nodes, four with 5–10 nodes, and seven small communities with less than 5 nodes. A total of 21 sub networks (right of **Figure 6**) were discovered from the gene division of Qi deficiency and blood stasis syndromes of stroke. Among them, there were nine large communities with more than 10 nodes, three with 5–10 nodes, and nine small communities with less than 5 nodes. We then calculated the GW values of the target syndromes (**Tables 3** and **4**) according to formula 4.

**Table 3:** Top rated genes GW values in Qi deficiency and blood stasis of coronary heart disease.

Id	Gene	Degree	Closeness	Betweeness	Hub	CMI	ZSW	GW
1	CHRNA4	1	1	0.29	1	1.29	1	2.29
2	BDNF	0.8	0.92	0.29	0.5	0.752174	1	1.75
3	CHRNB4	0.6	0.69	0	0.7	0.7	1	1.70
4	CHRNA5	0.5	0.62	0	0.64	0.64	1	1.64
5	CHRNB3	0.5	0.62	0	0.62	0.62	1	1.62
6	CHRNA6	0.4	0.62	0	0.54	0.54	1	1.54
7	COMT	0.5	0.77	0.14	0.44	0.530909	1	1.53
8	CHRNA3	0.5	0.62	0	0.52	0.52	1	1.52
9	CHRNB2	0.4	0.62	0	0.5	0.5	1	1.5
10	MAOA	0.4	0.77	0.14	0.38	0.452727	1	1.45

**Table 4:** Top rated genes GW values in Qi deficiency and blood stasis of stroke.

Id	Gene	Degree	Closeness	Betweeness	Hub	CMI	ZSW	GW
1	CXCR4	1	0.99	0.91	0.78	1.70	0.88	2.58
2	CXCL13	0.92	0.94	0.65	0.88	1.52	0.88	2.40
3	CCR7	0.85	0.88	0.27	1	1.06	0.88	1.94
4	CCL2	0.69	0.87	0.4	0.83	1.26	0.63	1.89
5	CXCL1	0.77	0.75	0.05	1.01	0.91	0.88	1.79
6	CCR4	0.62	0.87	0.16	0.93	0.85	0.88	1.73
7	CXCL9	0.62	0.75	0.04	0.88	0.85	0.88	1.73
8	CCL5	0.69	0.72	0.08	0.83	1.04	0.63	1.67
9	CCR2	0.54	0.72	0.02	0.87	0.75	0.88	1.63
10	CCR1	0.54	0.72	0.01	0.86	0.55	1	1.55

### Results of “Combination Method”

Since TCM diseases and syndromes are composed of a variety of symptoms, and these can be divided into primary and secondary symptoms. Under the guidance of TCM experts|, we clarified what symptoms are allocated to our two target syndromes, according to the criteria of TCM diagnostic, and following combined all target symptoms. By combining these main symptoms, the results can better reflect the essential characteristics of the target diseases.

1. Results of symptoms classification of Qi deficiency and blood stasis syndromes in CHD.
  - Main symptoms: chest pain, chest tightness.
  - Symptoms of Qi deficiency: shortness of breath, fatigue, perspiration, dizziness, cold limbs, insomnia, palpitation.
  - Blood stasis: light purple tongue body, hemiplegia, ecchymosis.
2. Results of symptoms classification of Qi deficiency and blood stasis syndrome in stroke; stroke
  - Main symptoms: hemiplegia, deviated mouth and/or tongue.
  - Qi deficiency: shortness of breath, fatigue, perspiration, pale complexion, dizziness, thirst, phlegm, white and greasy tongue coating.
  - Blood stasis: ecchymosis, red tongue body, numbness, irritability, constipation.

We firstly used Genclip software to obtain the genes corresponding to each symptom and their respective keywords. This was done according to the method in *Combination Method* (see **Table 5**). Unfortunately, no genes were related to “tongue disorder”, “insomnia” and “chest heaviness” by using Genclip. These three symptoms were found in Genclip, but since our aim was to analyze the combination, rather than individual symptoms removing these three aforesaid symptoms from the symptom combination would not impact our subsequent research.

**Table 5:** Number of genes discovered by keyword.

Id	Symptoms	Count
1	Dizziness	166
2	Constipation	210
3	Headache	338
4	Xerostomia	87
5	Irritability	122
6	Glossitis	19
7	Extremity Numbness	4
8	Hemiplegia	65
9	Sputums	4
10	Stroke	699
11	Respiratory Abnormality	76
12	Anemic	169
13	Lassitude	31
14	Ecchymoses	24
15	Sweating	129
16	Hemiplegia	65
17	Chest pain	225
18	Palpitation	49
19	Typhoid Fever	82
20	Chest Symptom Heaviness	0
21	Tongue Disorder	0
22	Insomnia	0

3. Symptom Combination Results of Qi Deficiency and Blood Stasis Syndromes in CHD

Using the formula combination of “main symptoms” + “Qi Deficiency” + “blood stasis” in Qi deficiency and blood stasis of CHD, 10 individual symptom combinations were obtained after deleting the keywords “Chest heaviness, insomnia and tongue disorder”.

Combination 1:
Chest pain + Respiratory abnormality + Palpitation + Hemiplegia
Combination 2:
Chest pain + Respiratory abnormality + Palpitation + Ecchymoses
Combination 3:
Chest pain + Lassitude + Palpitation + Hemiplegia
Combination 4:
Chest pain + Lassitude + Palpitation + Ecchymoses
Combination 5:
Chest pain + Sweating + Palpitation + Hemiplegia
Combination 6:
Chest pain + Sweating + Palpitation + Ecchymoses
Combination 7:
Chest pain + Dizziness + Palpitation + Hemiplegia
Combination 8:
Chest pain + Dizziness + Palpitation + Ecchymoses
Combination 9:
Chest pain + Typhoid fever + Palpitation + Hemiplegia
Combination 10:
Chest pain + Typhoid fever + Palpitation + Ecchymoses

4. Symptom Combination Results of Qi Deficiency and Blood Stasis Syndrome in Stroke

Using the formula combination of “main symptoms” + “Qi deficiency” + “blood stasis” in Qi deficiency and blood stasis in stroke, six different combinations of symptoms were obtained after deleting the keywords “Chest heaviness, insomnia and tongue disorder”.

Combination 1:
Hemiplegia + Stroke + Respiratory abnormality + Lassitude + Constipation + Xerostomia + Headache + Sputum + Ecchymoses + Extremity Numbness
Combination 2:
Hemiplegia + Stroke + Respiratory abnormality
+ Lassitude + Constipation + Xerostomia + Headache + Sputum + Glossitis + Irritability
Combination 3:
Hemiplegia + Stroke + Sweating + Constipation + Xerostomia + Headache + Sputum + Ecchymoses + Numb extremities
Combination 4:
Hemiplegia + Stroke + Sweating + Constipation + Xerostomia + Headache + Sputums + Glossitis + irritability
Combination 5:
Hemiplegia + Stroke + Anemic + Constipation + Xerostomia + Headache + Sputum + Ecchymoses + Numb extremities
Combination 6:
Hemiplegia + Stroke + Anemic + Constipation + Xerostomia + Headache + Sputum + Glossitis + Irritability

Comparing these combinations and removing any data repeated between them a total of 373 genes related to the syndrome of Qi deficiency and blood stasis of CHD and 804 genes relevant to the syndrome of Qi deficiency and blood stasis of stroke were obtained.

### Results Combination of “Decomposition” and “Combination”

According to the results of “decomposition method” and “combination method”, “decomposition method” had 84 genes related to the syndrome of Qi deficiency and blood stasis of CHD and 199 genes related to the syndrome of Qi deficiency and blood stasis of stroke, “combination method” had 373 genes related to the syndrome of Qi deficiency and blood stasis of CHD and 804 genes related to the syndrome of Qi deficiency and blood stasis of stroke. By matching the results of “decomposition method” and “combination method” a total of 84 genes related to Qi deficiency and blood stasis syndrome of CDH and 161 genes related to Qi deficiency and blood stasis syndrome of stroke were found.

**Figure 7:**
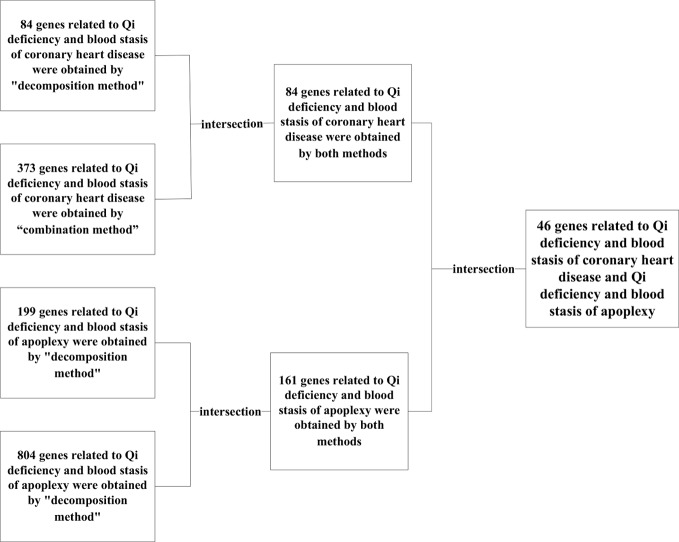
Gene integration steps.

Even though Qi deficiency and blood stasis syndrome of CHD is different from that of stroke, the same treatment can be used according to TCM. Since different diseases can have the same treatment there evidently should be similar pathological changes and pathogenic mechanisms between these diseases. Therefore, we decided to investigate the common genes of these two target syndromes (see **Figure 7**) for specific similarities. After comparison, 46 genes in both diseases were identified: CXCR4, HGF, EGF, BDNF, IL10, INS, IL12B, IL13, IGF2, IGF1R, CTNNB1, IFNG, HTR3A, EGFR, HLA-B, HLA-DQB1, HLA-DRB1, IFNGR1, IGF1, CRLF1, GDNF, FOXP3, AKT1, F3, BMP2, AR, CNTF, BCL2, IL6, COMT, CHRNA4, CLCF1, CNTFR, MET, MUC1, MAOA, LEP, CCK, IL4, BRCA1, C3, IL18, IL2, IL1B, INSR, IL17A.

We further analyzed the functions and biological pathways affected by the above 46 genes. An online software (https://www.kegg.jp/) was used to analysis the pathways of related genes (see **Table 6**).

**Table 6:** Recurring genes and pathways of target syndromes.

Channel Name	Genes	P Value
Inflammatory bowel disease (IBD)	IL4, IL17A, IL6, IL18, IFNG, IL13, IL12B, FOXP3, IFNGR1, IL10, IL2	2.49E-13
Cytokine-cytokine receptor interaction	LEP, IL4, IL17A, IL6, CNTF, CLCF1, CXCR4, IL18, IFNG, IL13, CNTFR, IL12B, IL10, IFNGR1, IL2	3.59E-13
HIF-1 signaling pathway	AKT1, EGFR, IGF1R, IL6, INS, BCL2, IFNG, IGF1, EGF, INSR, IFNGR1	3.07E-11
Jak-STAT signaling pathway	AKT1, IL4, LEP, IL6, CNTF, IFNG, IL13, CNTFR, IL12B, IFNGR1, IL10, IL2	5.4E-11
PI3K-Akt signaling pathway	AKT1, IL4, EGFR, IGF1R, IL6, INS, BCL2, MET, IGF1, HGF, EGF, INSR, BRCA1, IL2	3.34E-09
Prostate cancer	AKT1, EGFR, IGF1R, AR, INS, BCL2, IGF1, EGF, CTNNB1	7.76E-09
Pathways in cancer	AKT1, EGFR, IGF1R, AR, IL6, BMP2, CXCR4, BCL2, MET, IGF1, HGF, EGF, CTNNB1	2.36E-07
FoxO signaling pathway	AKT1, EGFR, IGF1R, IL6, INS, IGF1, EGF, INSR, IL10	2.66E-07
Rap1 signaling pathway	AKT1, EGFR, IGF1R, INS, MET, IGF1, HGF, EGF, INSR, CTNNB1	6.56E-07
Chagas disease (American trypanosomiasis)	AKT1, IL6, C3, IFNG, IL12B, IFNGR1, IL10, IL2	9.27E-07
Melanoma	AKT1, EGFR, IGF1R, MET, IGF1, HGF, EGF	1.04E-06
Tuberculosis	AKT1, IL6, C3, BCL2, IL18, IFNG, IL12B, IFNGR1, IL10	2.07E-06
Measles	AKT1, IL4, IL6, IFNG, IL13, IL12B, IFNGR1, IL2	3.82E-06
Proteoglycans in cancer	AKT1, EGFR, IGF1R, MET, IGF1, IGF2, IL12B, HGF, CTNNB1	4E-06
Focal adhesion	AKT1, EGFR, IGF1R, BCL2, MET, IGF1, HGF, EGF, CTNNB1	4.5E-06
Malaria	IL6, IL18, IFNG, MET, HGF, IL10	5.88E-06
Ras signaling pathway	AKT1, EGFR, IGF1R, INS, MET, IGF1, HGF, EGF, INSR	1.18E-05
Leishmaniasis	IL4, C3, IFNG, IL12B, IFNGR1, IL10	1.41E-05
African trypanosomiasis	IL6, IL18, IFNG, IL12B, IL10	2.45E-05
Allograft rejection	IL4, IFNG, IL12B, IL10, IL2	3.45E-05
Intestinal immune network for IgA production	IL4, IL6, CXCR4, IL10, IL2	6.3E-05
Toxoplasmosis	AKT1, BCL2, IFNG, IL12B, IFNGR1, IL10	0.00018
Glioma	AKT1, EGFR, IGF1R, IGF1, EGF	0.000249
AMPK signaling pathway	AKT1, LEP, IGF1R, INS, IGF1, INSR	0.000298
Adherens junction	EGFR, IGF1R, MET, INSR, CTNNB1	0.000448
Type I diabetes mellitus	INS, IFNG, IL12B, IL2	0.001164
Influenza A	AKT1, IL6, IL18, IFNG, IL12B, IFNGR1	0.001507
T cell receptor signaling pathway	AKT1, IL4, IFNG, IL10, IL2	0.001813
Endometrial cancer	AKT1, EGFR, EGF, CTNNB1	0.001956
Ovarian steroidogenesis	IGF1R, INS, IGF1, INSR	0.002195
Legionellosis	IL6, C3, IL18, IL12B	0.003836
Signaling pathways regulating pluripotency of stem cells	AKT1, IGF1R, BMP2, IGF1, CTNNB1	0.004221
Pertussis	IL6, C3, IL12B, IL10	0.006069
Asthma	IL4, IL13, IL10	0.006078
Salmonella infection	IL6, IL18, IFNG, IFNGR1	0.008094
Non-alcoholic fatty liver disease (NAFLD)	AKT1, LEP, IL6, INS, INSR	0.008759
Rheumatoid arthritis	IL17A, IL6, IL18, IFNG	0.009541
Progesterone-mediated oocyte maturation	AKT1, IGF1R, INS, IGF1	0.009847
Graft-versus-host disease	IL6, IFNG, IL2	0.013008
Herpes simplex infection	IL6, C3, IFNG, IL12B, IFNGR1	0.0136
Amoebiasis	IL6, IFNG, IL12B, IL10	0.01594
Oocyte meiosis	IGF1R, AR, INS, IGF1	0.018058
Aldosterone-regulated sodium reabsorption	INS, IGF1, INSR	0.018591
Insulin resistance	AKT1, IL6, INS, INSR	0.019401
Autoimmune thyroid disease	IL4, IL10, IL2	0.026985
Non-small cell lung cancer	AKT1, EGFR, EGF	0.027996
Regulation of lipolysis in adipocytes	AKT1, INS, INSR	0.03438
mTOR signaling pathway	AKT1, INS, IGF1	0.035495
Central carbon metabolism in cancer	AKT1, EGFR, MET	0.040091
Pancreatic cancer	AKT1, EGFR, EGF	0.041274
Fc epsilon RI signaling pathway	AKT1, IL4, IL13	0.041274
Renal cell carcinoma	AKT1, MET, HGF	0.04247
Transcriptional misregulation in cancer	IGF1R, IL6, MET, IGF1	0.042499
Colorectal cancer	AKT1, BCL2, CTNNB1	0.047383
ErbB signaling pathway	AKT1, EGFR, EGF	0.070363
Choline metabolism in cancer	AKT1, EGFR, EGF	0.088362
Systemic lupus erythematosus	C3, IFNG, IL10	0.088362
Toll-like receptor signaling pathway	AKT1, IL6, IL12B	0.094642

It can be seen from the above table that genes related to different diseases share certain common pathways. Nine shared signaling pathways (including AMPK, FoxO, ErbB, HIF-1, Jak-STAT, PI3K-Akt, Rap1, Ras, T cell receptor), 30 shared disease pathways (7 tumor related pathways, 13 infectious disease related pathways, 6 immune system disease related pathways, 4 endocrine disease pathways).

### Genes Related to Traditional Chinese Materia Medica

We searched the “TCM target database” of TCMIP v2.0 in order to further study the relevance of traditional Chinese materia medica to the target syndromes. Using the reverse search function of TCMIP v2.0 led us to the discovery of the medical components related to the 46 genes involved in our target syndromes.

**Figure 8:**
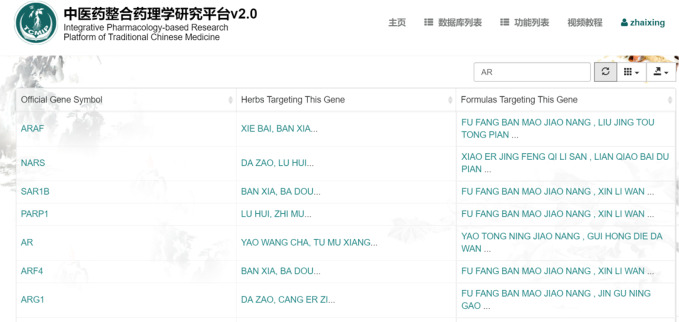
TCMIP v2.0 Query interface.

TCMIP v2.0 (Xu et al., 2019) is an intelligent data platform, which is embedded with a broad spectrum of authoritative algorithms including drugs physical and chemical properties, evaluation of drug composition, and prediction of drug targets. In addition, the system is also able to analyze functions and pathways related to drug targets and disease targets. This function allows for cross retrieval from traditional Chinese materia medica → formula → ingredients → target gene → function/pathway → disease. This cross search function can uncover the relevance of syndromes and drugs related to specific diseases, as well as rate the medicines' compatibility with target molecular groups. As shown in the **Figure 8**, a target gene was inserted into TCMIP v2.0 “TCM target database”. For example, insert “AR”, now the database will show the results of gene name, the specific genes corresponding to Chinese materia medica compounds and its corresponding prescription. With this search we found a total of 25 types of traditional Chinese materia medica related to the target syndromes;

Arestemona root, pinellia tuber, danshen root, common rush, adhesive rehmannia root, garden burnet root, barbary wolfberry fruit, seaweed extract, puncturevine caltrop fruit, Indian Quassiawood twing and leaf, lotus seed, pyrola herb, European verbena herb, dwarf lilyturf tuber, hogfennel root, ginseng, mulberry twig, common yam rhizome, inula root, longstamen onion bulb, inula flower, yanhusuo, Yào Wáng; Chá, poppy shell, and milkwort root.

## Discussions

According to TCM CHD belongs to the category of chest arthralgia, palpitation, and subjective fatigue, it is located in the heart and blood vessels and mostly caused by the invasion of external evils, internal injury of emotions, improper diet, stress, and deficiency of viscera. In the development of CHD, Qi deficiency is the main factor, with inseparable bonds to phlegm and blood stasis (Houde, 2017).*Yilin Gaicuo* said “deficiency of vital energy(Qi) will lead to Qi not reaching the blood vessels, blood vessels without Qi will accumulate and form blood stasis”, the etiology and pathogenesis of cardiovascular disease are mostly related to Qi deficiency and blood stasis (Jianqi and Yu, 2016), a vast amount of TCM literature point out that, according to TCM Qi deficiency and blood stasis are the two main pathological changes causing CHD. Stroke is located in the brain, and is according to TCM closely related to the heart, liver, spleen and kidney. Wind, fire, phlegm, blood stasis, deficiency and toxin are the main pathogenic factors of stroke (Zhe, 2010). Wang Qingren believed that the root of hemiplegia induced by stroke could be found in the deficiency of vital energy (Qi), based on this theory, he composed the *Buyang Huanwu Decoction* for the treatment of Qi deficiency and blood stasis syndromes of stroke (Zhengtai et al., 2017).

Our study shows that Qi deficiency and blood stasis of CHD and stroke includes some inflammatory factors: IL-10, FOXP3, cell apoptosis, differentiation, and proliferation. Cell apoptosis, differentiation, and proliferation: BCL-2, AKT1, CLCF1, HGF, EGF, IGF1, CXCR4, and CTNNB1. Neurotrophic factors includes; CNTF, BDNF, and GDNF. Our results verify that the inflammatory responses, apoptosis, proliferation, and other mechanisms are shared by the two target syndromes.

Interleukin is involved in regulating the activation, proliferation, inflammatory response, and differentiation of immune cells. Interleukin-10 (IL-10) plays an important role in the pathogenesis of CHD, acute cerebral infarction, and other diseases (Chao et al., 2013; Dong et al., 2015). FoxP3 regulates T-cell specific transcription factor that can secrete IL-10 as well as regulate immune functions and cause immune tolerance (Huehn et al., 2009). Other research has found that for coronary artery disease patients with low IL-10/TNF-α ratio has low left ventricular ejection fraction, with high incidence of cardiac related diseases within 10 years. It is therefore speculated that the imbalance of IL-10 and TNF α may be relevant to the pathological development of atherosclerosis and heart failure (Dopheide et al., 2015).

The Bcl-2 gene is involved in apoptosis. Some scholars (Zhongfu et al., 2001) believe that oxygen free radicals and calcium excess can induce apoptosis during myocardial ischemia-reperfusion. Bcl-2 can inhibit apoptosis by interaction with antioxidants, inhibiting the production of oxygen free radicals, changing the cell outflow of Ca2^+,^ and by regulating cGMP. Hepatocyte growth factor (HGF), insulin-like growth factor (IGF), epidermal growth factor (EGF), and other cytokines can activate PI3-Akt, Jak-STAT, and other signal pathways that are active in the facilitation of cell differentiation, angiogenesis, and apoptosis inhibition (Hosui et al., 2006). Cardiac nutrition literacy cytokine (CLCF1) is mainly involved in the Jak-STAT signaling pathway, cytokine, and chemokine mediated signaling pathway and negative regulation of apoptosis (Elsaeidi et al., 2014). Chemokine receptor (CXCR4) promotes cell proliferation. It has been suggested that up regulation of CXCR4 signaling pathway may be an important mechanism in the treatment of CHD (Hristov et al., 2007). CTNNB1 is a β-catenin. The stable expression of β-catenin in cells can up-regulate the survival rate of regulatory T cells, induce the apoptosis of non-conditional T cells, accelerate cell cycles and promote cell proliferation (Ding et al., 2008).

Neurotrophic factors can induce, regulate and control the survival, growth, and migration of neurons as well as establish functional connections with other cells by regenerating axons as part of nerve regeneration (Lichtman, 1987). As an important neurotrophic factor, glial cell-derived neurotrophic factor (GDNF) can reduce the area of infarction during the acute stage of stroke (Gunther et al., 2005). The increased expression of brain-derived neurotrophic factor (BDNF) may be relevant to the recovery of neural function and plasticity after cerebral ischemia (Ergul et al., 2012).

**Figure 9:**
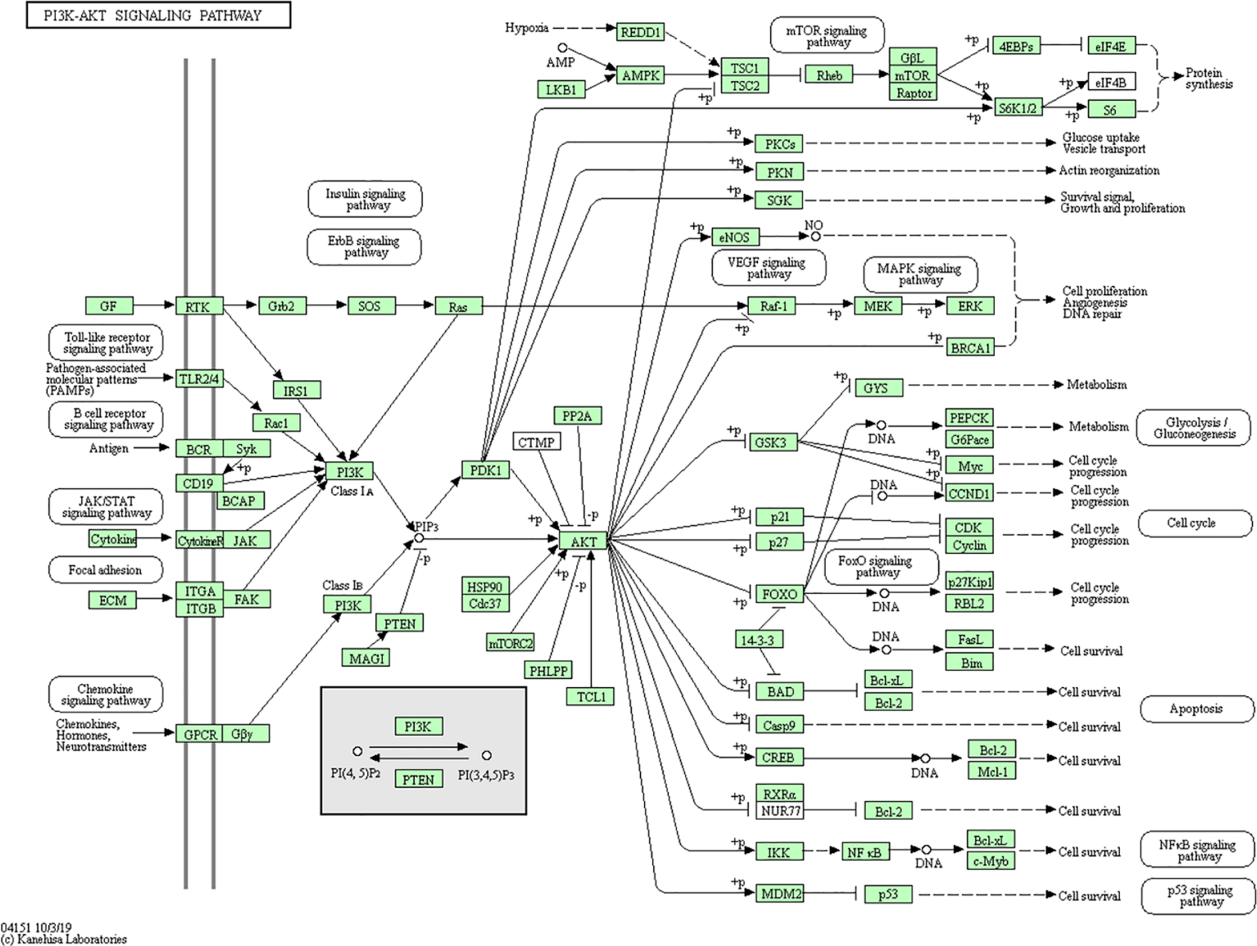
PI3K-Akt signal pathways.

By searching the KEGG database, we further confirmed the interrelations of the above mentioned genes and their pathways. PI3K-Akt signal path (**Figure 9**) involves FoxO, ErbB, Ras, and other sub modules, while FoxO, ErbB, HIF-1, and Jak-STAT also contain signals of the sub module PI3K-Akt. It is known that PI3K-Akt signaling pathway can promote endothelial regeneration, vasodilation, and platelet adhesion in cardiomyocyte, hence improve their survival rate and functionality (PAN et al., 2017). In addition, PI3K-Akt signaling pathway is also involved in cerebral infarction and other diseases (Zhang Hong and Junjian, 2011).

We found, through our analysis that the traditional Chinese herbs related to the 46 specific genes includes; Qi replenishing herbs *ginseng* and *yam*; blood activating herb *danshen* root; resuscitation herb *milkwort* root; phlegm dissolving herbs *pinellia* tuber, *hogfennel* root, and inula flower. This indicated that Qi replenishing, blood activating, resuscitation, and phlegm dissolving herbs have close connection to Qi deficiency and blood stasis syndrome. This discovery is consistent with TCM theory and clinical practice. Some scholars (Mengna et al., 2017) think that CHD is caused by Qi deficiency, Yang deficiency, blood deficiency, and Yin deficiency with Qi deficiency as the common denominator. Qi is the driving force of blood circulation. When Qi flows, blood flows. Weak Qi leads to blood not flowing smoothly that in turn leads to stagnation and pain. Qi deficiency, phlegm coagulation and blood stasis constitute the key pathogenesis of CHD and stroke. In the treatment of these diseases, emphasis should be given on Qi and blood circulation in order to tonify the body and resolve blood stasis. Modern pharmacological research validates that ginseng contains *ginsenoside*, ginseng polysaccharide, volatile oil, and other components. These chemicals can regulate the heart function, blood vessels, blood pressure, and central nervous system. *Ginsenoside* as the main component of ginseng has proven *in vivo* to protect cardiomyocytes, improve myocardial metabolism, and to increase stroke volume (Wang et al., 2015). *Ginsenoside* Rb1 prevents lipopolysaccharide induced cardiomyocyte inflammation by inhibiting the PI3K/Akt signaling pathway (Zhiyang et al., 2018). Tanshinone, *Danshensu* and other water-soluble components in *danshen* root can improve myocardial metabolism, increase coronary blood flow, and reduce the incidence of myocardial ischemia and myocardial infarction. *Jiawei Danshen Yin* (ruoxia et al., 2020) can activate the PI3K/Akt signaling pathway through negative regulation of PTEN that in turn inhibit apoptosis of myocardial cells during ischemia-reperfusion and improve the survival rate of myocardial cells during ischemia and hypoxia. This leads to improvements of the heart and reduces the injury of cardiovascular disease to the heart. Pinellia tuber can regulate blood lipid metabolism leading to improved hemodynamics, reduce blood viscosity, and inhibition of RBC aggregation (Wenyue et al., 2002).

This study preliminarily clarified that the pathological mechanism of Qi deficiency and blood stasis is involved in cell necrosis, apoptosis and inflammatory responses. As well as involving FoxO, ErbB, HIF-1, Jak-STAT, PI3K-Akt signal pathways. We aim to reveal the molecular mechanism based on biological research to provide a scientific basis for the TCM theory of “treating different diseases with the same method”.

## Summary And Prospect

Using Qi deficiency and blood stasis as our fundamental concepts to analyze the extensive online literature and big data with “decomposition” model, “combination” model, “information extraction,” and “complex network”. Ultimately, leading to discussions on the biological basis of the TCM theory “treating different diseases with the same method”. This study provides a bridge between TCM and modern medicine, and also demonstrates the use of information technology to study the TCM term “syndrome”.

It is a new approach to use big data and complex network as a method to analysis the biological basis of the TCM concept “treating different diseases with the same method”. Follow up research will be needed to verify and validate the genes related to the two target syndromes as well as the practicality of our method. We plan to conduct more in-depth researches on the relationship between traditional Chinese materia medica—genes–syndromes–diseases by exploring their common molecular mechanism. Then, we will get a deeper biological understanding of the TCM concept “treating different diseases with the same method”.

## Data Availability Statement

The datasets presented in this study can be found in online repositories. The names of the repository/repositories and accession number(s) can be found in the article/**Supplementary Material**.

## Author Contributions

XZ: Put forward research ideas and write papers. XW: Paper revision. LW: thesis writing. WW: Thesis writing. XP: Thesis writing. LX: Put forward research ideas and write papers.

## Funding

This work was supported by the Fundamental Research Funds for the Central Universities (2020-JYB-ZDGG-070, 2019-JYB-JS-031) and the National Natural Science Foundation of China (No. 81603499).

## Conflict of Interest

The authors declare that the research was conducted in the absence of any commercial or financial relationships that could be construed as a potential conflict of interest.

The reviewer YW declared a shared affiliation, with no collaboration, with the authors to the handling editor at the time of the review.

## References

[B1] Artzy-Randrup Y. Fleishman S. J. Ben-Tal N. Stone L. (2004). Comment on “Network motifs: simple building blocks of complex networks” and “Super families of evolved and designed networks”. Science 305 (5687), 1107. 10.1126/science.1099334 15326338

[B2] Bin S. (2002). Overview of information extraction technology. Terminology Standardization Inf. Technol. 3 (1), 28–32.

[B3] Bunescu R. Mooney R. (2005a). “Subsequence kernels for relation extraction A,” in In Proceedings of NIPS C], vol. 2005 (Vancouver Canada: MIT Press), 171–178.

[B4] Chao Y Tong C. Kexia Z. (2013). Correlation between serum IL-6 and carotid atherosclerosis in patients with acute cerebral infarction. J. Modern Lab. Med. 28 (2), 152–153,156. 10.3969/j.issn.1671-7414.2013.02.049

[B5] Deng M. Tu Z. Sun F. Chen T. (2004). Mapping Gene Ontology to proteins based on protein-protein interaction data. Bioinformatics. 20 (6), 895–902. 10.1093/bioinformatics/btg500 14751964

[B6] Ding S. S. Hong S. H. Wang C. Guo Y. Wang Z. K. Xu Y. (2013). Acupuncture modulates the Neuro-Endocrine-Immune network. QJM 107 (5), 341–345. 10.1093/qjmed/hct196 24106314

[B7] Ding Y. Shen S. Lino A. C. (2008). Stable expression of β-catenin promotes regulatory T cell survival and induces non conditional T cell apoptosis. Chin. J. Tumor Biother. (02), 168.

[B8] Dong G. Xiaodong W. Guangyi J. Wenqi J. (2015). Research progress in the structure of Toll like receptor ligand complex. J. Cell Mol. Immunol. 31 (4), 553–556.

[B9] Dopheide J. F. Knopf P. Zeller G. C. Vosseler M. Abegunewardene N. Münzel T. (2015). Low IL-10/TNF-α Ratio in Patients with Coronary Artery Disease and Reduced Left Ventricular Ejection Fraction with a Poor Prognosis After 10 Years. Inflammation 38 (2), 911–922. 10.1007/s10753-014-0053-5 25384561

[B10] Elsaeidi F. Bemben M. A. Zhao X. F. Goldman D. (2014). Jak/Stat signaling stimulates zebrafish optic nerve regeneration and overcomes the inhibitory actions of Socs3 and Sfpq. Neurosci 34 (7), 2632–2644. 10.1523/JNEUROSCI.3898-13.2014 PMC3921430 24523552

[B11] Ergul A. Alhusban A. Fagan S. C. (2012). Angiogenesis: a harmonized target for recovery after stroke. Stroke 43 (8), 2270–2274. 10.1161/STROKEAHA.111.642710 22618382 PMC3404267

[B12] Fan D. Qianru Z. Yuanjia H Yitao W. (2014). Study on the mechanism of Buyang Huanwu Decoction in the prevention and treatment of Qi deficiency and blood stasis based on network analysis. Chin. J. Traditional Chin. Med. 39 (22), 4418–4425. 10.4268/cjcmm20142227 25850278

[B13] Gunther A. Kuppers-Tiedt L. Schneider P. M. (2005). Reduced infarct volume and differential effects on glial cell activation after hyperbaric oxygen treatment in rat permanent focal cerebral ischaemia. Eur. J. Neurosci. 21 ( 11), 3189–3194. 10.1111/j.1460-9568.2005.04151.x 15978027

[B14] Hosui A. Takehara T. Ohkawa K. Kanazawa Y. Tatsumi T. Yamaguchi S. (2006). Suppressive effect on hepatocyte differentiation of hepatitis C virus core protein. Biochem. Biophys. Res. Commun. 346 (4), 1125–1130. 10.1016/j.bbrc.2006.05.114 16806084

[B15] Houde R. (2017). Treatment of cardiovascular diseases from the perspective of integration of Chinese and Western Medicine. Clin. Res. Traditional Chin. Med. 9 (10), 145–146. 10.3969/j.issn.1674-7860.2017.10.070

[B16] Hristov M. Zernecke A. Bidzhekov H. Liehn E. A. Shagdarsuren E. Ludwig A. (2007). Importance of CXC chemokine receptor 2 in the homing of human peripheral blood endothelial progenitor cells to sites of arterial injury. Circ. Res. 100 (4), 590–597. 10.1161/01.RES.0000259043.42571.68 17272812

[B17] Huang Z. X. Tian H. Y. Hu Z. F. Zhou Y. B. Zhao J. Yao K. T. (2008). GenCLiP: a software program for clustering gene lists by literature profiling and constructing gen eco-occurrence networks related to custom keywords. BMC Bioinf. 13 (9), 308. 10.1186/1471-2105-9-308 PMC2483997 18620599

[B18] Huehn J. Polansky J. K. Hamann A. (2009). Epigenetic control of FOXP3 expression: the key to a stable regulatory T-cell lineage? Nat. Rev. Immunol. 9 (2), 83–89. 10.1038/nri2474 19114986

[B19] Isaković K. Janković B. D. (1973). Neuro-Endocrine Correlates of Immune Response. Int. Arch. Allergy Immunol. 45 (3), 373–384. 10.1159/000231055 4126543

[B20] Jianqi L Yu S. (2016). Research progress on the prevention and treatment of cardiovascular diseases by the method of Supplementing Qi and activating blood circulation. J. Guangxi Univ. Traditional Chin. Med. 19 (01), 78–80. 10.7501/j.issn.0253-2670.2015.10.027

[B21] Lamurias A. Clarke L. A. Couto F. M. (2017). Extracting microRNA-gene relations from biomedical literature using distant supervision. PloS One 12 (3), e0171929. 10.1371/journal.pone.0171929 28263989 PMC5338769

[B22] Lan W. Yusheng Z. (1990). The role of doubtful nucleus in the regulation of cellular immunity by acupuncture at Zusanli. J. OVeterinary Univ. 10 (2), 146–148.

[B23] Li S. Zhang Z. Q. Wu L. J. Zhang X. G. Li Y. D. Wang Y. Y. (2007). Understanding ZHENG in traditional Chinese medicine in the context of neuro-endocrine -immune network. Syst. Biology IET 1 (1), 51–60. 10.1049/iet-syb:20060032 17370429

[B24] Lichtman J. W. Taghert P. H. (1987). Developmental neurobiology.Trophic factor theory matures. Nature 326 (6111), 336. 10.1038/326336a0 3031503

[B25] Lim J. Hao T. Shaw C. Patel A. J. Szabó G. Rual J.-F. (2006). A protein-protein interaction network for human inherited ataxias and disorders of Purkinje cell degeneration. Cell 125 (4), 801–814. 10.1016/j.cell.2006.03.032 16713569

[B26] Liu J. Zhai X. Liao X. (2019). Bibliometric analysis on cardiovascular disease treated by traditional Chinese medicines based on big data. Int. J. Parallel Emergent Distributed Syst. 1, 1–17 10.1080/17445760.2019.1606912

[B27] Ma T. Tan C. Zhang H. Wang M. Q. Ding W. J. Li S. (2010). Bridging the gap between traditional Chinese medicine and systems biology: the connection of Cold Syndrome and NEI network. Mol. Biosyst. 6 (4), 613–619. 10.1039/b914024g 20237638

[B28] Mengna J. Li Y. Zetao C. (2017). Professor Shao nianfang's experience in the treatment of coronary heart disease with the method of Invigorating Qi and promoting blood circulation. J. Guangxi Univ. Traditional Chin. Med. (3), 020(003), 5–6. 10.3969/j.issn.2095-4441.2017.03.003

[B29] Ono T. Hishigaki H. Tanigami A. Takagi T. (2001). Automated extraction of information on protein-protein interactions from the biological literature. Bioinformatics 17 (2), 155–161. 10.1093/bioinformatics/17.2.155 11238071

[B30] Pan Y. Yin J. Cai X. M. Li L. Xu Y. Yu C. (2017). Research progress on interven-tion of traditional hinese medicine on coronary heart diseasethrough PI3K / Akt signaling pathway. China Tradit Herb Drugs 48 (19), 4100–4104. 10.7501/j.issn.0253-2670.2017.19.030s

[B31] Ruoxia W. Zhengyang L. Weisi B. Wenyao Z. Jing L. Xiang W. (2020). Study on the inhibitory effect of Jiawei Danshen drink on IRI through PTEN / PI3K / Akt signal pathway. Hunan J. Traditional Chin. Med. 36 (03), 137–140. 10.16808/j.cnki.issn1003-7705.2020.03.058

[B32] Shao L. (2007). Computational system biology of traditional Chinese medicine and cold heat syndrome. World Sci. Technol. - Modernization Traditional Chin. Med. 9 (1), 105–111. 10.11842/wst.2007.1

[B33] Wang R. X. He R. L. Jiao H. X. Dai M. Mu Y.-P. Hu Y. (2015). Ginsenoside Rb1 attenuates agonist-induced contractile response via inhibition of store-operated calcium entry in pulmonary arteries of normal and pulmonary hypertensive rats. Cell Physiol. Biochem. 35 (4), 1467–1481. 10.1159/000373966 25791507

[B34] Wenyue J. Yu Y. Yanyan L. (2002). Effects of pinellia, Guawei, Fritillaria thunbergii and Acorus tatarinowii on Hemorheology in rats. J. Traditional Chin. Med. 43 (3), 215–225.

[B35] Wu Y. Zhang F. Yang K. Fang S. Bu D. Li H. (2018). SymMap: an integrative database of traditional Chinese medicine enhanced by symptom mapping. Nucleic Acids Res. 47, D1110–D1117. 10.1093/nar/gky1021 PMC6323958 30380087

[B36] Xiao J. Su J. Zhou G. D. Tan C. L. (2005). “Protein-protein interaction extraction: a supervised learning approach A,” in Proceedings of the First International Symposium on Semantic Mining in Biomedical C (Turku, Finland: SMBM), 51–59.

[B37] Xiao-yu ZHENG (2002). Guidance Principle of Clinical Study on NewDrug of Traditional Chinese Medicine (Beijing: Chinese Medical and Pharmaceutical Science Press).

[B38] Xing Z. Xuanchao F. Jingwei L. Gao K. Jia Z. Zhao H. (2015). Neuro-Endocrine-Immune Biological Network Construction Of Qi Deficiency Pattern And Qi Stagnation Pattern In Traditional Chinese Medicine. J. Biol. Syst. 23 (2), 305–321. 10.1142/S0218339015500163

[B39] Xu H. Y. Zhang Y. Q. Liu Z. M. Chen T. Lv C. Y. Tang S. H. (2019). ETCM: An encyclopaedia of traditional Chinese medicine. Nucleic Acids Res. 47 (D1), D976–D982. 10.1093/nar/gky987 30365030 PMC6323948

[B40] Xuezhong Z. Zhaohui W Baoyan L. (2004). Knowledge Diseovery in Biomedieal Literature:Survey and ProsPeet. Complex Syst. ComPlexity Seienee 1, 3, 1–3.

[B41] YIDH CHENGH (2017). A Research on Comprehensive Measurement Of Complex Network Structure Relationship Based on Social Network Analysis. Stat. Decision (7), 14–17. 10.13546/j.cnki.tjyjc.2017.07.003

[B42] Zhang Hong S. Junjian Z. (2011). Research progress of PI3K / Akt signaling pathway in nervous system diseases. Med. Rev. 17 (18), 2732–2735.

[B43] Zhe Z. (2010). Preliminary discussion on the evolution of stroke pathogenesis. World Traditional Chin. Med. 5 (1), 3–4. 10.3969/j.issn.1673-7202.2010.01.002

[B44] Zhengtai Z. Shihuan T. Yuexiang M. (2017). Research progress of Buyang Huanwu Decoction in treating stroke of Qi deficiency and blood stasis type. Shandong J. Traditional Chin. Med. 36 (1), 71–73. 10.16295/j.cnki.0257-358x.2017.01.024

[B45] Zhiyang Z. ran G lulu Y. (2018). Ginsenoside Rb1 protects lipopolysaccharide induced myocardial inflammatory response by inhibiting PI3K / Akt signaling pathway. Chin. J. Evidence Based Cardiovasc. Med. 10 (07), 823–826. 10.3969/j.issn.1674-4055.2018.07.13

[B46] Zhongfu M Hong M. Rengao Y. Bin L. Quansheng Z. Jinlang W. (2001). Study on apoptosis of myocardial cells in elderly patients with acute myocardial infarction. Chin. J. Gerontol. (01), 6–9. 10.3969/j.issn.1005-9202.2001.01.003

